# Racial and ethnic disparities in COVID-19 booster vaccination among U.S. older adults differ by geographic region and Medicare enrollment

**DOI:** 10.3389/fpubh.2023.1243958

**Published:** 2023-08-10

**Authors:** Kaleen N. Hayes, Daniel A. Harris, Andrew R. Zullo, Preeti Chachlani, Katherine J. Wen, Renae L. Smith-Ray, Djeneba Audrey Djibo, Ellen P. McCarthy, Alexander Pralea, Tanya G. Singh, Cheryl McMahill-Walraven, Michael S. Taitel, Yalin Deng, Stefan Gravenstein, Vincent Mor

**Affiliations:** ^1^Center for Gerontology and Healthcare Research, Brown University School of Public Health, Providence, RI, United States; ^2^Department of Health Services, Policy, and Practice, Brown University School of Public Health, Providence, RI, United States; ^3^Department of Epidemiology, Brown University School of Public Health, Providence, RI, United States; ^4^Center of Innovation in Long-Term Services and Supports, Providence Veterans Affairs Medical Center, Providence, RI, United States; ^5^Department of Medicine, Health, and Society, Vanderbilt University, Nashville, TN, United States; ^6^Walgreens Center for Health and Wellbeing Research, Walgreen Company, Deerfield, IL, United States; ^7^CVS Health, Safety Surveillance and Collaboration, Blue Bell, PA, United States; ^8^Hinda and Arthur Marcus Institute for Aging Research, Hebrew SeniorLife, Boston, MA, United States; ^9^Division of Gerontology, Department of Medicine, Beth Israel Deaconess Medical Center, Harvard Medical School, Boston, MA, United States; ^10^Division of Geriatrics, Warren Alpert Medical School of Brown University, Providence, RI, United States

**Keywords:** mRNA vaccines, COVID-19, 2019-nCoV vaccine, healthcare disparities, aged, mRNA-1273, BNT162b2 vaccine, booster vaccine

## Abstract

**Introduction:**

COVID-19 booster vaccines are highly effective at reducing severe illness and death from COVID-19. Research is needed to identify whether racial and ethnic disparities observed for the primary series of the COVID-19 vaccines persist for booster vaccinations and how those disparities may vary by other characteristics. We aimed to measure racial and ethnic differences in booster vaccine receipt among U.S. Medicare beneficiaries and characterize potential variation by demographic characteristics.

**Methods:**

We conducted a cohort study using CVS Health and Walgreens pharmacy data linked to Medicare claims. We included community-dwelling Medicare beneficiaries aged ≥66 years who received two mRNA vaccine doses (BNT162b2 and mRNA-1273) as of 8/1/2021. We followed beneficiaries from 8/1/2021 until booster vaccine receipt, death, Medicare disenrollment, or end of follow-up (12/31/2021). Adjusted Poisson regression was used to estimate rate ratios (RRs) and 95% confidence intervals (CIs) comparing vaccine uptake between groups.

**Results:**

We identified 11,339,103 eligible beneficiaries (mean age 76 years, 60% female, 78% White). Overall, 67% received a booster vaccine (White = 68.5%; Asian = 67.0%; Black = 57.0%; Hispanic = 53.3%). Compared to White individuals, Black (RR = 0.78 [95%CI = 0.78–0.78]) and Hispanic individuals (RR = 0.72 [95% = CI 0.72–0.72]) had lower rates of booster vaccination. Disparities varied by geographic region, urbanicity, and Medicare plan/Medicaid eligibility. The relative magnitude of disparities was lesser in areas where vaccine uptake was lower in White individuals.

**Discussion:**

Racial and ethnic disparities in COVID-19 vaccination have persisted for booster vaccines. These findings highlight that interventions to improve vaccine uptake should be designed at the intersection of race and ethnicity and geographic location.

## 1. Introduction

Due to their effectiveness at reducing the risk of severe illness and death from COVID-19 (1, 2), booster vaccinations represent an essential public health intervention that may be integrated into annual vaccination programs (3). There were racial and ethnic disparities in the uptake of the primary COVID-19 vaccine series (4–6). Several social and structural factors contribute to these differences, including vaccine hesitancy and varying access to vaccines (7–10). Despite attempts through federal and local policies to promote vaccine equity (11), racial and ethnic disparities in booster uptake likely remain (5). Efforts to identify and reduce racial and ethnic differences in COVID-19 booster vaccine uptake should be ongoing, especially due to continued disparities in SARs-CoV-2 infection and higher COVID-19-associated mortality in Black and Hispanic populations (12, 13).

Research is needed to understand whether racial and ethnic disparities are greater or lesser within certain subgroups, such as those defined by age, sex, and geographic location. Understanding the extent to which disparities vary by these factors can help to inform public health interventions designed to reduce racial and ethnic disparities in booster vaccine uptake. Prior studies have documented substantial variation in the likelihood of vaccination and vaccine hesitancy across counties in the US, as well as by race and ethnicity, yet few studies have examined how disparities in uptake differ by geographic location and other relevant factors, such as type of health insurance coverage (14, 15). Characterizing heterogeneity of disparities within these subgroups can provide useful insight into what may drive differences in vaccine uptake and identify targets for public health interventions.

Our aim was to identify potential racial and ethnic differences in booster vaccine receipt in a large cohort of U.S. Medicare beneficiaries and to explore variation in those differences by age, sex, geographic region, type of Medicare plan (i.e., Medicare Advantage [MA] or Fee-for-Service [FFS]), and dual eligibility status for Medicaid. Our specific study objective was to estimate the relative rate of COVID-19 booster vaccine receipt between race and ethnicity groups among Medicare beneficiaries in 2021.

## 2. Materials and methods

### 2.1. Study data sources, design and population

We conducted a retrospective cohort study using pharmacy data from CVS Health and Walgreens linked to Medicare claims. Medicare is the U.S. federal health insurance program that provides coverage to people aged 65 years or older and select people younger than age 65 who have certain disabilities or end-stage renal disease (16). An orientation on the use of Medicare administrative data for observational research are available (17). The database, named COVVAXAGE, includes all CVS Health and Walgreens pharmacy customers at any time between 2018 and 2021 who were 65 years or older and had a pharmacy record paid by Medicare. Pharmacy customers who were Medicare beneficiaries were matched deterministically to the 100% Medicare Enrollment File based on name, address, and date of birth. COVVAXAGE has been used in prior studies of COVID-19 vaccine safety and effectiveness in older adults (18). Approximately 95% of eligible customers were matched, creating a cohort of over 38 million individuals overall and more than 27 million individuals aged 65 years or older as of January 1, 2021 (representing 70% of Medicare beneficiaries 65+) (16). COVVAXAGE links complete Medicare enrollment information, inpatient claims, outpatient and provider (i.e., carrier) claims, and CVS Health and Walgreens pharmacy records.

Our study population included community-dwelling Medicare beneficiaries aged ≥66 years with 2 doses of an mRNA vaccine (BNT162b2 or mRNA-1273) documented in Medicare carrier and outpatient files or pharmacy vaccination records between January 1 and August 1, 2021. We excluded individuals who died or were not enrolled in Medicare as of or before the start of follow-up (August 1, 2021). We then excluded those who were in institutional long-term or post-acute care or did not have 12 months of continuous enrollment in Medicare FFS or MA as of the start of follow-up. Finally, we excluded individuals who had a history of three or more mRNA vaccine doses or a Johnson and Johnson vaccine, as the likelihood for mRNA booster vaccination was potentially different for these individuals during the study follow-up period versus those with 2 doses of the primary series of the mRNA vaccines. We restricted the study population to individuals ≥66 to ensure a one-year lookback in Medicare data for covariates, as age-entitled Medicare enrollment begins at age 65.

### 2.2. Race and ethnicity

Race and ethnicity was the primary independent variable of interest, defined using the Research Triangle Institute (RTI) race code that provided the following mutually exclusive categories: non-Hispanic White (henceforth “White”), Black, Asian/Pacific Islander (henceforth “Asian”), Hispanic, American Indian/Alaska Native (henceforth Native American), Other Race/Ethnicity, and Missing Race/Ethnicity (19, 20). As the RTI measure poorly identifies Native American ethnicity and “other” races and ethnicities (21), we focused our analysis on comparing vaccine uptake between White (reference group), Black, Asian, and Hispanic individuals. However, individuals belonging to other racial and ethnic groups were included in all models, with their estimates of booster vaccine uptake reported in the supplement.

### 2.3. COVID-19 booster vaccination

The outcome was receipt of a third dose of a COVID-19 vaccine (henceforth “booster vaccine”), which was defined using Common Procedural Terminology (CPT) codes or relevant pharmacy records (definitions available in Supplementary Table S1). Individuals were followed from August 1, 2021 until receipt of a booster vaccine, or they were censored upon death, disenrollment from Medicare, or end of follow-up (December 31, 2021). We did not censor on entry into long-term or post-acute care as we anticipated that the rate of new admission during the five-months of follow-up would be low and non-differential between race/ethnicity groups. The follow-up period for the primary analysis ended on December 31, 2021 because of an administrative change in COVID-19 vaccine billing to Medicare for MA beneficiaries that began on January 1, 2022 (22). However, as a sensitivity analysis, we restricted the population to FFS beneficiaries and extended follow-up for booster vaccine uptake to May 15, 2022 (latest available data at the time of analysis). As some individuals likely received a booster vaccine prior to their official eligibility based on the time since their second dose, we did not apply any time-based restrictions on when booster vaccines could be ascertained based on CDC recommendations during the study period. Finally, we followed individuals on a calendar time scale (i.e., starting on August 1, 2021) rather than a vaccine exposure-anchored time scale (i.e., from the day after completion of their primary vaccine series) to estimate rates of vaccine uptake that only included follow-up time in which booster vaccines were available to patients (in or after August 2021).

### 2.4. Covariates

To describe the population, we collected demographic (e.g., age, sex, and zip code of residence) and clinical information inclusive of and before the start of follow-up. Geographic region of residence was defined using the US Department of Health and Human Services (HHS) Regions, which are categorized by the major US city in that particular region (e.g., Boston) (23). An area-level measure of social deprivation was obtained from the American Community Survey (2015–2019) (24). We used the National Center for Health Statistics urban–rural classification scheme to categorize counties as large central metropolitan counties (i.e., inner cities), large fringe metropolitan (i.e., suburbs), medium metropolitan, small metropolitan, micropolitan, and non-core. Metropolitan areas are considered urban whereas micropolitan and non-core areas are considered rural. Medicare enrollment type was defined as a categorical variable based on the combination of an individual’s Medicare insurance (FFS, MA, or a mix of FFS/MS) and Medicaid (supplemental insurance for low-income individuals) dual eligibility status in the 12 months prior to follow-up (e.g., dual Medicaid eligible and FFS; not dual Medicaid eligible and FFS).

Finally, using all available FFS Medicare claims (inpatient, outpatient, carrier, etc.) in the 12 months prior to the start of follow up, we measured the following covariates: comorbidities were defined using Hierarchical Condition Categories (HCC) codes (25); frailty was measured using the Kim cumulative deficit claims-based frailty index (CFI) (26); and comorbidity burden was measured using the Combined Comorbidity Index (27, 28). Clinical information was only captured among FFS beneficiaries because MA encounter data (used to derive clinical covariates) was unavailable for the study period at the time of analysis. This clinical information was used only for descriptive purposes and not included in regression models used for the final analysis.

### 2.5. Primary comparison of interest and analysis

We plotted the cumulative incidence of booster vaccination with 95% confidence intervals (CIs) for each group using the Kaplan–Meier estimator. The cumulative incidence at the end of follow-up corresponded to the incidence of booster vaccination over the average follow-up time of 15.5 weeks. Generalized linear models with a Poisson distribution and log link function estimated rate ratios (RR) and 95% CIs for booster vaccination across racial and ethnic groups. Models included a log-transformed offset for follow-up time and covariates for age, sex, and geographic region. To test the robustness of results from the primary analyses to alternate model specifications in a sensitivity analysis, we included a 4-level covariate combining type of Medicare insurance and dual Medicaid eligibility (i.e., FFS or MA with or without dual Medicaid enrollment) (29).

### 2.6. Variation in disparities by subgroups

To describe potential variation in racial and ethnic differences in booster vaccination by age, sex, geographic region, degree of urbanicity, and Medicare plan/Medicaid eligibility, we first plotted cumulative incidence curves for each group. As a formal assessment of effect measure modification on the multiplicative scale and to obtain subgroup-specific estimates of booster vaccine uptake between race/ethnicity groups, we fit a series of adjusted Poisson models with statistical interaction product terms between race and ethnicity group and each sociodemographic characteristic of interest (e.g., race/ethnicity*region) – specifying separate models for each interaction being assessed. Two-sided *p*-values for homogeneity were calculated, and values <0.05 were considered indicative of heterogeneity in differences. Interacted models were adjusted for the other sociodemographic characteristics as in the primary analyses (age, sex, geographic region). All analyses were conducted in SAS v9.4 (SAS Institute, Cary NC) and Stata version 17.0 (StataCorp LLC, College Station, TX).

### 2.7. Ethics approval

Brown University’s Institutional Review Board approved the study with a waiver of informed consent (Protocol # 2103002950). We established data use agreements with CVS Health, Walgreens, and the Medicare & Medicaid Resource Information Center (MedRIC) for Centers for Medicare and Medicaid (CMS) data files.

## 3. Results

We identified 11,339,103 eligible beneficiaries for study inclusion (mean age 76.2 [SD 7.4] years, 60% female, 78% White, 7.5% Black, 6.8% Hispanic, 4.1% Asian; Table 1; Supplementary Table S2 presents all measured characteristics). Supplementary Figure S1 contains a flow diagram of study exclusions. Over a mean of 15.5 (SD 5.4) weeks of follow up, 66.6% of the cohort received a booster vaccine (Kaplan Meier cumulative incidence estimate), 4.2% died (4.5, 3.8 3.3, and 2.6% of White, Black, Hispanic, and Asian patients, respectively), and < 0.02% disenrolled before the end of follow-up (events and censoring stratified by race/ethnicity are available in Supplementary Table S3). Supplementary Figure S2 shows Kaplan Meier cumulative uptake of booster vaccination by sex, age, geographic region, and Medicare/Medicaid enrollment.

**Table 1:** Characteristics of community-dwelling Medicare beneficiaries 66 years and older with 2 documented mRNA vaccine doses as of August 1, 2021.^a^

Characteristics	Overall^a^	White race	Black race	Asian race	Hispanic ethnicity
Unique beneficiaries, *n* (%)	11,339,103 (100.0%)	8,863,202 (100.0%)	855,319 (100.0%)	461,650 (100.0%)	773,790 (100.0%)
Age in years, mean (SD)	76.20 (7.36)	76.51 (7.47)	75.04 (6.85)	75.97 (7.31)	75.71 (6.94)
Age in years, *n* (%)					
66–69	2,643,312 (23.31%)	1,987,430 (22.42%)	237,473 (27.76%)	111,807 (24.22%)	188,880 (24.41%)
70–74	3,288,099 (29.00%)	2,481,305 (28.00%)	263,453 (30.80%)	136,167 (29.50%)	229,065 (29.60%)
75–79	2,292,169 (20.21%)	1,827,399 (20.62%)	163,665 (19.13%)	91,065 (19.73%)	160,065 (20.69%)
80–84	1,532,112 (13.51%)	1,236,918 (13.96%)	104,921 (12.27%)	61,677 (13.36%)	105,951 (13.69%)
85–89	933,860 (8.24%)	772,809 (8.72%)	55,443 (6.48%)	35,843 (7.76%)	58,061 (7.50%)
90+	649,551 (5.73%)	557,341 (6.29%)	30,364 (3.55%)	25,091 (5.44%)	31,768 (4.11%)
Female sex, *n*(%)	6,746,330 (59.50%)	5,280,685 (59.58%)	554,109 (64.78%)	270,119 (58.51%)	468,629 (60.56%)
Geographic region (23), *n*(%)					
Boston	785,317 (6.93%)	678,063 (7.65%)	24,735 (2.89%)	20,879 (4.52%)	27,635 (3.57%)
New York	1,281,419 (11.30%)	873,281 (9.85%)	81,177 (9.49%)	66,442 (14.39%)	209,633 (27.09%)
Philadelphia	1,045,382 (9.22%)	856,749 (9.67%)	110,113 (12.87%)	27,883 (6.04%)	17,970 (2.32%)
Atlanta	2,226,798 (19.64%)	1,737,900 (19.61%)	290,320 (33.94%)	32,485 (7.04%)	110,504 (14.28%)
Chicago	2,419,196 (21.33%)	2,095,549 (23.64%)	143,936 (16.83%)	46,195 (10.01%)	53,306 (6.89%)
Dallas	927,542 (8.18%)	648,520 (7.32%)	106,915 (12.50%)	27,819 (6.03%)	118,475 (15.31%)
Kansas City	429,773 (3.79%)	387,007 (4.37%)	22,583 (2.64%)	4,647 (1.01%)	5,561 (0.72%)
Denver	200,250 (1.77%)	179,310 (2.02%)	2,315 (0.27%)	2,936 (0.64%)	8,746 (1.13%)
San Francisco	1,639,651 (14.46%)	1,067,698 (12.05%)	61,024 (7.13%)	220,747 (47.82%)	213,640 (27.61%)
Seattle	303,383 (2.68%)	270,147 (3.05%)	3,730 (0.44%)	10,966 (2.38%)	7,358 (0.95%)
Missing	80,392 (0.71%)	68,978 (0.78%)	8,471 (0.99%)	651 (0.14%)	962 (0.12%)
Social deprivation index (24), *n* (%)					
Quintile 1 (low deprivation)	2,213,826 (19.52%)	1,959,358 (22.11%)	46,729 (5.46%)	74,134 (16.06%)	43,199 (5.58%)
Quintile 2	2,230,557 (19.67%)	1,937,061 (21.86%)	70,958 (8.30%)	77,678 (16.83%)	64,135 (8.29%)
Quintile 3	2,178,134 (19.21%)	1,819,937 (20.53%)	101,818 (11.90%)	94,930 (20.56%)	86,036 (11.12%)
Quintile 4	2,208,971 (19.48%)	1,758,184 (19.84%)	169,682 (19.84%)	86,494 (18.74%)	125,931 (16.27%)
Quintile 5 (high deprivation)	2,240,698 (19.76%)	1,284,306 (14.49%)	448,075 (52.39%)	124,535 (26.98%)	318,690 (41.19%)
Missing	266,917 (2.35%)	104,356 (1.18%)	18,057 (2.11%)	3,879 (0.84%)	135,799 (17.55%)
Urbanicity, *n* (%)					
Large central metro	3,084,935 (27.21%)	2,056,587 (23.20%)	352,136 (41.17%)	228,372 (49.47%)	330,496 (42.71%)
Large fringe metro	3,240,539 (28.58%)	2,655,316 (29.96%)	209,295 (24.47%)	123,226 (26.69%)	136,585 (17.65%)
Medium metro	2,605,362 (22.98%)	2,134,496 (24.08%)	166,955 (19.52%)	87,958 (19.05%)	127,535 (16.48%)
Small metro	1,018,903 (8.99%)	895,694 (10.11%)	54,580 (6.38%)	11,348 (2.46%)	29,395 (3.80%)
Micropolitan	802,856 (7.08%)	709,225 (8.00%)	41,954 (4.91%)	9,291 (2.01%)	18,515 (2.39%)
Non-core	457,209 (4.03%)	409,048 (4.62%)	30,195 (3.53%)	1,351 (0.29%)	5,350 (0.69%)
Missing	129,299 (1.14%)	2,836 (0.03%)	204 (0.02%)	104 (0.02%)	125,914 (16.27%)
Medicare and Medicaid enrollment (29)					
FFS with no Medicaid dual enrollment	5,706,585 (50.33%)	4,917,746 (55.48%)	270,478 (31.62%)	147,836 (32.02%)	162,221 (20.96%)
FFS with Medicaid dual enrollment	352,430 (3.11%)	176,434 (1.99%)	41,808 (4.89%)	61,932 (13.42%)	56,116 (7.25%)
MA with no Medicaid dual enrollment	4,426,011 (39.03%)	3,301,840 (37.25%)	408,265 (47.73%)	167,787 (36.35%)	415,436 (53.69%)
MA with Medicaid dual enrollment	403,358 (3.56%)	151,294 (1.71%)	75,391 (8.81%)	61,617 (13.35%)	102,746 (13.28%)
MIXED with no Medicaid dual enrollment	381,712 (3.37%)	286,171 (3.23%)	45,447 (5.31%)	13,522 (2.93%)	23,945 (3.09%)
MIXED with Medicaid dual enrollment	69,007 (0.61%)	29,717 (0.34%)	13,930 (1.63%)	8,956 (1.94%)	13,326 (1.72%)
History of comorbidities (25)^b^					
Cancer	953,327 (15.73%)	814,447 (15.99%)	51,868 (16.61%)	24,659 (11.76%)	28,413 (13.01%)
Chronic Obstructive Pulmonary Disease	640,770 (10.58%)	560,042 (10.99%)	34,117 (10.92%)	12,991 (6.19%)	19,232 (8.81%)
Congestive Heart Failure	765,781 (12.64%)	648,310 (12.73%)	51,271 (16.42%)	19,384 (9.24%)	28,110 (12.87%)
Diabetes	1,498,904 (24.74%)	1,154,815 (22.67%)	129,745 (41.55%)	75,568 (36.02%)	83,877 (38.42%)
Ischemic Stroke	177,035 (2.92%)	146,733 (2.88%)	13,137 (4.21%)	5,520 (2.63%)	6,949 (3.18%)
Major Organ Transplant	22,189 (0.37%)	17,884 (0.35%)	1,426 (0.46%)	753 (0.36%)	1,130 (0.52%)
Vascular disease	1,174,801 (19.39%)	1,000,709 (19.64%)	68,260 (21.86%)	31,134 (14.84%)	42,846 (19.62%)
Renal Conditions	350,354 (5.78%)	288,327 (5.66%)	28,990 (9.28%)	10,145 (4.84%)	14,101 (6.46%)
Claims-based frailty index (26)^b^					
Non-frail	3,336,657 (55.07%)	2,771,470 (54.40%)	161,265 (51.64%)	134,691 (64.21%)	116,279 (53.26%)
Pre-Frail	2,329,921 (38.45%)	1,980,852 (38.88%)	129,803 (41.57%)	67,480 (32.17%)	87,189 (39.93%)
Frail	392,437 (6.48%)	341,858 (6.71%)	21,218 (6.79%)	7,597 (3.62%)	14,869 (6.81%)
Combined Comorbidity Index, mean (SD) (27, 28)^b^	1.73 (2.65)	1.73 (2.65)	2.10 (2.98)	1.41 (2.47)	1.81 (2.75)

FFS, Fee-for-Service; MA, Medicare Advantage; MIXED, Mixed FFS/MA enrollment in lookback period; SD, Standard Deviation.

a Beneficiaries with Native American, Other Race/Ethnicity, or Missing race/ethnicity presented in the Supplement.

b Measured for FFS Beneficiaries only (*n* = 6,059,015) due to incomplete claims data for MA beneficiaries to capture comorbidities. Prevalence may differ for MA beneficiaries.

### 3.1. Racial and ethnic disparities in booster vaccination

**Figure 1:**
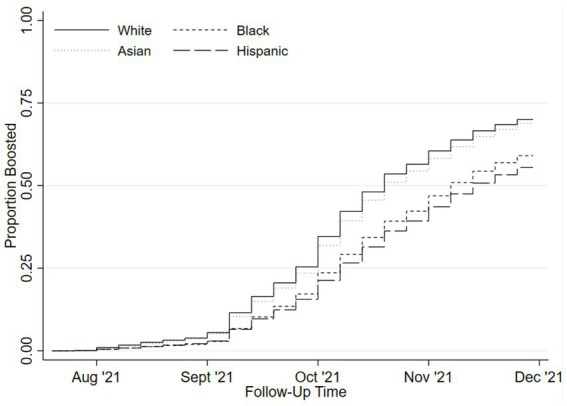
Cumulative incidence of COVID-19 booster vaccinations by race and ethnicity.

We observed significant differences in the cumulative incidence of booster vaccination by race and ethnicity by December 31, 2021 (Kaplan Meier cumulative incidence estimates: White = 68.5%; Asian = 67.0%; Black = 57.0%; Hispanic = 53.3%, Figure 1) by the end of follow up. After controlling for age, sex, and region in regression models, Black individuals (RR = 0.78 [95%CI = 0.78–0.78]) and Hispanic individuals (RR = 0.72 [95% = CI 0.72–0.72]) had a significantly lower rate of booster vaccination compared to White individuals. Asian individuals had a slightly lower rate of booster vaccination compared to White individuals (RR = 0.97 [95%CI = 0.97–0.98]). Unadjusted, partially-adjusted, and fully-adjusted model results are available in Supplementary Table S4. Results from the sensitivity analysis including Medicare/Medicaid enrollment type were concordant with the primary results (Supplementary Table S5), except that after adjusting for enrollment, Asian beneficiaries were more likely to receive a booster vaccination (RR = 1.08 [95% CI (1.07–1.08)]). Finally, when we restricted the study population to FFS beneficiaries and extended follow-up to May 15, 2022, the overall proportion of those with a booster vaccination increased (Kaplan Meier cumulative incidence for follow-up to December 2021 vs. May 2022 for FFS beneficiaries: 69% vs. 76%; Supplementary Figure S3). Relative rates of booster vaccination between race and ethnicity groups were similar with this extended follow-up in the FFS population, though disparities were modestly lessened (e.g., Black versus White beneficiaries: RR = 0.78 changed to RR = 0.82; model results available in Supplementary Table S6).

### 3.2. Variation in racial and ethnic disparities in booster uptake by other sociodemographic characteristics

**Figure 2:**
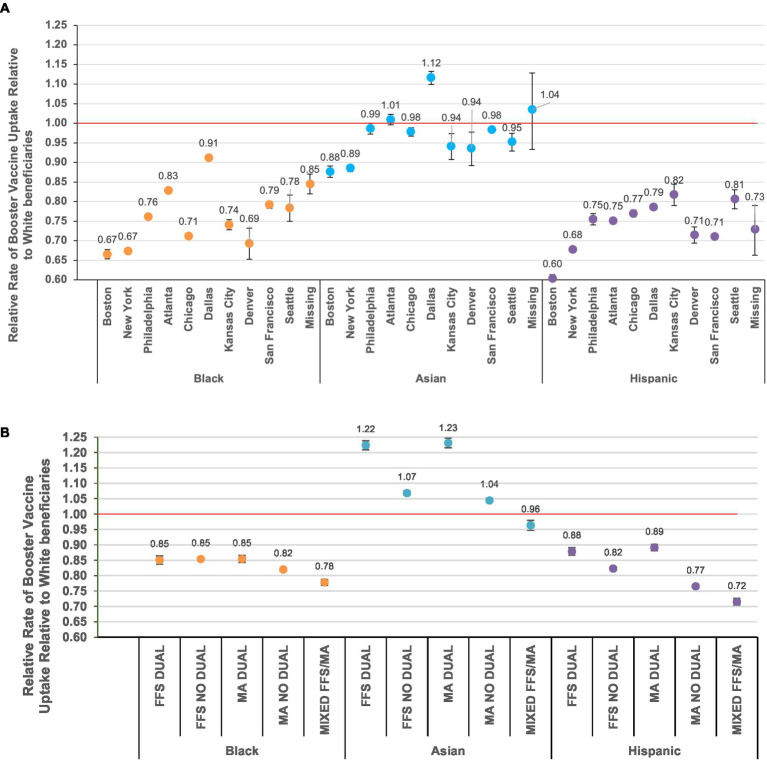
Relative rates of booster uptake* with 95% confidence intervals compared to White beneficiaries by race and ethnicity, stratified by **(A)** U.S. Dept. of Health and Human Services geographic region (23) and **(B)** Medicare and Medicaid enrollment. “No dual”, not dual eligible for Medicaid enrollment (29); FFS, Fee-for-Service; MA, Medicare Advantage. *Rate ratios (RRs) estimated from Poisson regression models with an interaction term for race/ethnicity group * (geographic region or enrollment category) to output group-specific estimates. NB: figures depicting uptake between groups by sex, age, and urbanicity are presented in the supplement.

Little variation in the degree of racial and ethnic differences was observed between sex and age subgroups; however, estimates varied widely between regions, degrees of urbanicity, and types of Medicare enrollment and Medicaid eligibility (Figure 2; Supplementary Figures S4, S5; Supplementary Table S7). For example, in the Boston region, Black individuals showed 34% lower relative booster uptake compared to White individuals (RR = 0.66; 95%CI = 0.65–0.68); however, in the Dallas region, Black individuals showed a 9% lower relative booster vaccination rate than White individuals (RR = 0.91; 95%CI = 0.90–0.92; homogeneity *p* < 0.001) (Figure 2A). Similar patterns were observed across levels of urbanicity, with larger racial and ethnic differences in booster vaccination in the most urban counties than in the most rural counties (e.g., Black vs. White race among those living in large central metropolitan counties = 0.70 [95%CI = 0.69–0.70]; Black vs. White race among those living in non-core counties = 0.91 [95%CI = 0.90–0.93]) (Supplementary Figure S5).

The magnitude of observed relative rates in booster vaccination between racial and ethnic groups when stratified by geographic location was largely the result of changes in the absolute vaccine uptake in White individuals; the degree of absolute uptake in Black and Hispanic individuals varied to a lesser degree. For example, in the Boston region, 73% of White individuals received a booster vaccine compared to 55% of Black individuals; however, in the Dallas region 61% of White individuals received a booster vaccine compared to 58% of Black individuals (Supplementary Table S7). The range of absolute booster uptake across regions was greatest for White individuals (maximum = 73% [Boston region]; minimum = 61% [Dallas]) and smallest for Black individuals (maximum = 58% [San Francisco]; minimum = 51% [Denver]).

Wide variation in booster vaccination across race and ethnicity was observed by type of Medicare enrollment and Medicaid eligibility (Figure 2B; Supplementary Table S7). For example, among those who switched between FFS and MA in the year prior to the start of follow-up, after adjustment, Black individuals had 0.78 times the rate of booster vaccination compared to White individuals (95%CI = 0.77–0.79). However, racial and ethnic differences were generally smaller among those with dual Medicaid eligibility (e.g., Hispanic vs. White among those with FFS and dual Medicaid eligibility = 0.88 [95%CI = 0.87–0.89]; Hispanic vs. White among those with FFS and no dual Medicaid eligibility = 0.82 [95%CI = 0.82–0.83]). Uptake of booster vaccines was much lower among those with versus without dual eligibility overall, and similar to the geography-based analyses, the degree of racial and ethnicity differences was largely a result of lower vaccine uptake in White individuals (Supplementary Table S7).

## 4. Discussion

In this cohort of over 11 million Medicare beneficiaries, we identified racial and ethnic disparities in booster vaccine uptake, with Black (57%) and Hispanic (53%) individuals having consistently lower booster vaccine receipt than White individuals (69%). The relative magnitude of these disparities varied markedly across geographic regions, level of urbanicity, and type of Medicare enrollment. Notably, the magnitude of the observed racial and ethnic disparities reflected in relative rates of uptake depended largely on the level of vaccination among White beneficiaries, thereby demonstrating the value of reporting absolute measures of vaccine uptake alongside relative comparisons.

Some of the observed racial and ethnic disparities are consistent with those identified in initial COVID-19 vaccine receipt, while others differ. As with the primary vaccine series, Black and Hispanic beneficiaries experienced disparities, though booster vaccine disparities appear greater than the primary series (i.e., 12 and 15% lower cumulative uptake of the booster vaccine for Black and Hispanic beneficiaries, respectively, versus-7 and + 5% for the primary series) (4). As others have noted, several factors, including differential access to certain types of vaccination sites (e.g., mass vaccination clinics, mobile vaccinations, physician’s offices) may contribute to these differences in uptake (30). Highlighting receipt of booster vaccinations *via* pharmacies, which Medicare beneficiaries accessed approximately twice as often as physician’s offices prior to the COVID-19 pandemic (31) and whose services have been show to increase immunization rates (32) and improve other health outcomes (33, 34) is one potential strategy to increase booster vaccination uptake. Vaccine hesitancy may contribute to disparities in uptake, yet Black and White individuals had comparable vaccination intentions in December 2020, and thereafter belief in the benefits of the vaccine increase among Black individuals at a higher rate than White individuals (30).

Interestingly, we observed large differences in booster vaccination receipt according to individuals’ geographic location, including urbanicity, and their racial and ethnic group, but relatively small differences by sex and age group. Our findings align with prior theories that link geographic location, political identity, and race to vaccine hesitancy and one’s likelihood of vaccination (15). Relative differences in booster vaccination between Black, Hispanic, and White individuals were greatest in the Boston and New York regions, as well as more urban areas, largely due to higher absolute rates of vaccination in White individuals. From the most rural to most urban counties, absolute rates of vaccination for White individuals increased monotonically from 61 to 70%; however, the proportion of vaccinated Black individuals across rural–urban levels varied between 55 and 59%.

Finally, we also observed smaller relative racial and ethnic disparities among those dually eligible for enrollment in Medicaid - an income-based healthcare supplement. CMS and the Biden Administration released guidance just prior to the availability of booster vaccines that aimed to increase uptake of COVID-19 vaccines through increasing reimbursement for vaccine administration and adding payment for counseling on COVID-19 vaccinations for Medicaid-enrolled individuals (35). Although relative racial and ethnic disparities in booster vaccination were smaller among those eligible for dual Medicaid enrollment, this group generally showed the lowest absolute uptake compared to those in traditional Medicare. Therefore, efforts to expand and evaluate interventions to increase uptake among those dually enrolled may need to be expanded or modified.

Our study has limitations to note. Firstly, the RTI race/ethnicity codes used in this study are accurate for White and Black beneficiaries (91–99% agreement with other data sources) (21). However, this measure is less sensitive for people of Asian race or Hispanic ethnicity, and has very low accuracy for Native American and other groups (21). Moreover, the use of mutually exclusive and limited racial and ethnicity categories in the RTI race code restricts our ability to identify certain groups (e.g., Hispanic Black). Misclassification of race and ethnicity information can result in inaccurate capture of outcomes among Medicare beneficiaries (36); beneficiaries with misclassified or over-simplified race/ethnicity data may be at even higher risk of disparities than their accurately classified counterparts (37). Work to continuously monitor the validity of race/ethnicity data in Medicare (38) and make race/ethnicity measures derived from improved algorithms, like the Medicare Bayesian Improved Surname Geocoding algorithm (39, 40), available to researchers is imperative to improve healthcare research for a broader range of racial and cultural identities. Secondly, as with other data sources, we could not include beneficiaries who received their primary vaccine series through a mass vaccination site or other location if the vaccine was not billed to Medicare or captured through pharmacy records. Nevertheless, we were able to identify over 11 million Medicare beneficiaries who were eligible for a booster dose of the COVID-19 vaccine from which inferences about uptake among all Medicare beneficiaries may be made.

In conclusion, we identified racial and ethnic disparities in COVID-19 booster vaccination uptake among a large population of Medicare beneficiaries, with Black and Hispanic individuals experiencing the greatest differences in vaccination. These disparities varied widely across geographic regions, and to a lesser degree, type of Medicare and Medicaid enrollment. As such, tailoring access to booster vaccines, potentially by emphasizing vaccination availability at pharmacies, will likely help to mitigate racial and ethnic disparities in vaccination, thereby contributing to improved health outcomes related to COVID-19 (7–10). A next step to reduce inequalities in COVID-19 vaccination and infections is to develop and test the effectiveness of tailored interventions to increase booster vaccination uptake.

## Data availability statement

The data analyzed in this study is subject to the following licenses/restrictions: investigators must establish data use agreements with CVS Health, Walgreens, and the Medicare & Medicaid Resource Information Center (MedRIC) for Centers for Medicare and Medicaid (CMS) to access these data files. Investigators with funded or who have in-progress funding applications to the National Institute on Aging who are interested in learning more about these data should contact Vincent Mor (vincent_mor@brown.edu) or Kaleen Hayes (kaley_hayes@brown.edu).

## Ethics statement

The studies involving humans were approved by Brown University Institutional Review Board. The studies were conducted in accordance with the local legislation and institutional requirements. The ethics committee/institutional review board waived the requirement of written informed consent for participation from the participants or the participants' legal guardians/next of kin because of the use of secondary data sources.

## Author contributions

KH: conceptualization, formal analysis, methodology, project administration, visualization, investigation, supervision, writing – original draft, and writing – review & editing. DH: conceptualization, methodology, project administration, and writing – review & editing. AZ and VM: conceptualization, methodology, funding acquisition, supervision, and writing – review & editing. PC: conceptualization, methodology, formal analysis, and writing – reviewing & editing. KW: conceptualization, methodology, and writing – review & editing. DD: methodology, data curation, and writing – review & editing. RS-R: methodology, data curation, writing – review & editing, and supervision. MT, TS, and CM-W: data curation, writing – review & editing, and supervision. YD and AP: conceptualization and writing – review & editing. EM and SG: conceptualization, methodology, funding acquisition, and writing – review & editing. All authors contributed to the article and approved the submitted version.

## Funding

This work was supported by the National Institute of Aging (NIA) of the National Institutes of Health under Award Number U54AG063546, which funds NIA Imbedded Pragmatic Alzheimer’s Disease and AD-Related Dementias Clinical Trials Collaboratory (NIA IMPACT Collaboratory). Supplemental funding was provided under grant numbers U54AG063546-S07 and U54AG063546-S08. The content is solely the responsibility of the authors and does not necessarily represent the official views of the National Institutes of Health.

## Conflict of interest

KH has received grant funding paid directly to Brown University for collaborative research from Insight Therapeutics and Sanofi for research on complex insulin regimens. KH also serves as a consultant for the Canadian Agency for Drugs and Technologies in Health. AZ has received grant funding paid directly to Brown University by Sanofi for collaborative research on the epidemiology of infections and vaccinations among nursing home residents and infants. SG is a recipient of support from the Pfizer to study pneumococcal vaccines and from Sanofi and Seqirus to study influenza vaccines. SG also performs consulting work for Icosavax, Janssen, Merck, Moderna, Novavax, Pfizer, Sanofi, Seqirus, and Vaxart; has served on the speaker’s bureaus for Seqirus, Janssen and Sanofi; and was paid to chair data safety monitoring boards from Longeveron and SciClone. RS**-**R, MT, and TS are employees of Walgreens and have received funding from Moderna and Pfizer to study vaccine uptake and effectiveness. DD and CM-W are full-time employees of CVS Health and conduct work for government, public, and private organizations, including pharmaceutical companies, as part of their employment.

The remaining authors declare that the research was conducted in the absence of any commercial or financial relationships that could be construed as a potential conflict of interest.

## Publisher’s note

All claims expressed in this article are solely those of the authors and do not necessarily represent those of their affiliated organizations, or those of the publisher, the editors and the reviewers. Any product that may be evaluated in this article, or claim that may be made by its manufacturer, is not guaranteed or endorsed by the publisher.
