# Supplementary material for: Racial and ethnic disparities in COVID-19 booster vaccination among U.S. older adults differ by geographic region and Medicare enrollment
Source: Front Public Health. 2023 Aug 10;11:1243958. doi: 10.3389/fpubh.2023.1243958 (PMC10456997; doi:10.3389/fpubh.2023.1243958)
Supplement: Supplementary file 1 [file Table_1.docx]

Supplementary Material

Variation in racial and ethnic disparities in COVID-19 booster vaccination among older adults differs by geographic region and Medicare enrollment

**Kaleen N. Hayes^1,2*^, Daniel A. Harris^1,2^, Andrew R. Zullo^1,2,3,4^, Preeti Chachlani^1,2^, Katherine J. Wen^1,2,9^, Renae L. Smith-Ray^5^, Djeneba Audrey Djibo^6^, Ellen P. McCarthy^8,9^, Alexander Pralea^1^, Tanya G. Singh^5^, Cheryl McMahill-Walraven^6^, Michael S. Taitel^5^, Yalin Deng^2^, Stefan Gravenstein^1,2,4,10^, Vincent Mor^1,2,4^**

^1^Center for Gerontology and Healthcare Research, Brown University School of Public Health, Providence, RI, USA

^2^Department of Health Services, Policy, and Practice, Brown University School of Public Health, Providence, RI, USA

^3^ Department of Epidemiology, Brown University School of Public Health, Providence, RI, USA

^4^ Center of Innovation in Long-Term Services and Supports, Providence Veterans Affairs Medical Center, Providence, RI, USA

^5^ Walgreens Center for Health & Wellbeing Research, Walgreen Company, Deerfield, IL, United States

^6^CVS Health Clinical Trial Services, Safety, Surveillance & Collaboration, Blue Bell, PA

^7^Vanderbilt University, Department of Medicine, Health, and Society, Nashville, TN, United States

^8^Hinda and Arthur Marcus Institute for Aging Research, Hebrew SeniorLife, Boston, MA

^9^Division of Gerontology, Department of Medicine, Beth Israel Deaconess Medical Center, Harvard Medical School, Boston, MA, United States

^10^Division of Geriatrics, Warren Alpert Medical School of Brown University, Providence, Rhode Island, United States

**Table of Contents**

**Supplemental Figure 1.** Study Cohort Exclusions Diagram.

**Supplemental Figure 2**. Kaplan-Meier cumulative incidence of booster vaccination by a) age group, b) sex, c) geographic region*, and d) Medicare/Medicaid enrollment.

**Supplemental Figure 3.** Kaplan-Meier cumulative incidence of booster vaccination when restricting to Medicare Fee-for-Service beneficiaries and extending follow-up to May 15, 2022 (n=6,059,015).

**Supplemental Figure 4**. Relative rates with 95% confidence intervals of booster uptake by race and ethnicity, stratified by a) sex and b) age group.

**Supplemental Figure 5.** Relative rates with 95% confidence intervals of booster uptake by race and ethnicity stratified degree of urbanicity.

**Supplementary Table 1.** Common Procedural Terminology (CPT) Codes used to Identify COVID-19 Vaccines.

**Supplemental Table 2.** Characteristics of all community-dwelling Medicare beneficiaries 66 years and older with 2 documented mRNA vaccine doses as of August 1, 2021.

**Supplemental Table 3.** Number of beneficiaries with booster vaccine receipt, death, and censoring events by race and ethnicity group.

**Supplemental Table 4.** Results from crude, partially-adjusted, and fully-adjusted models comparing receipt of booster vaccinations by race and ethnicity.

**Supplemental Table 5.** Results from sensitivity analysis including Medicare and Medicaid enrollment type in the regression models comparing booster vaccine receipt.

**Supplemental Table 6.** Results from sensitivity analysis restricting to Fee-for-Service Medicare beneficiaries and extending follow-up to May 15, 2022 (n=6,059,015).

**Supplemental Table 7.** Absolute booster vaccine uptake by race and ethnicity group across region, urbanicity, and Medicare enrollment.

# Supplementary Figures

## Supplemental Figure 1. Study Cohort Exclusions Diagram.


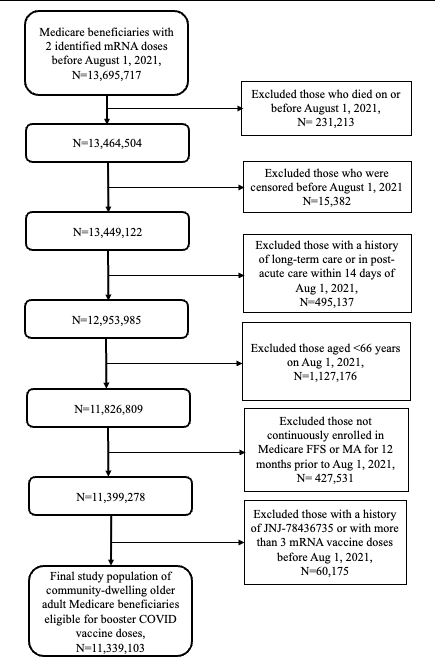


## Supplemental Figure 2. Kaplan-Meier cumulative incidence of booster vaccination by a) age group, b) sex, c) geographic region*, and d) Medicare/Medicaid enrollment.

a)


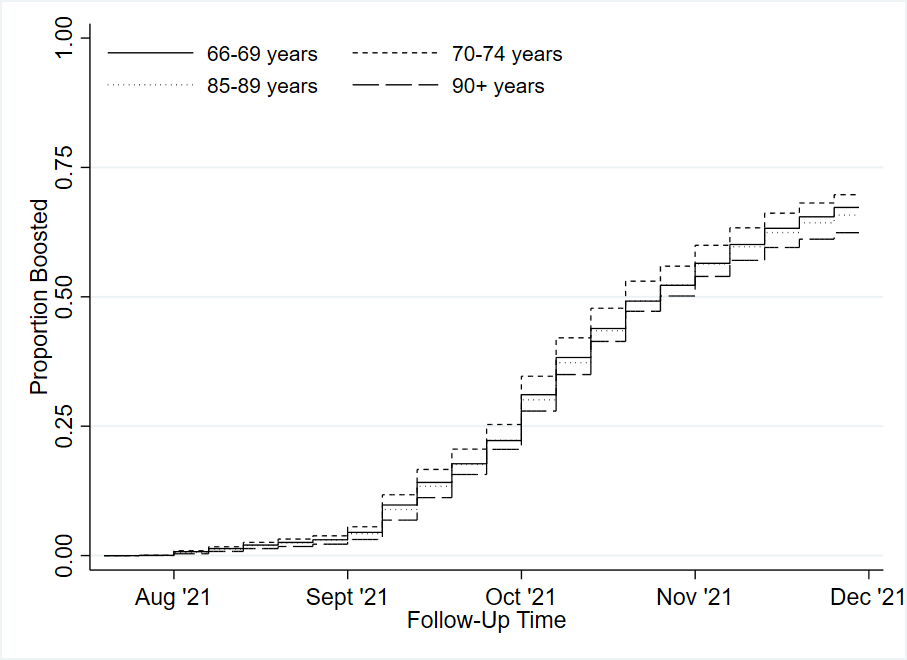


b)


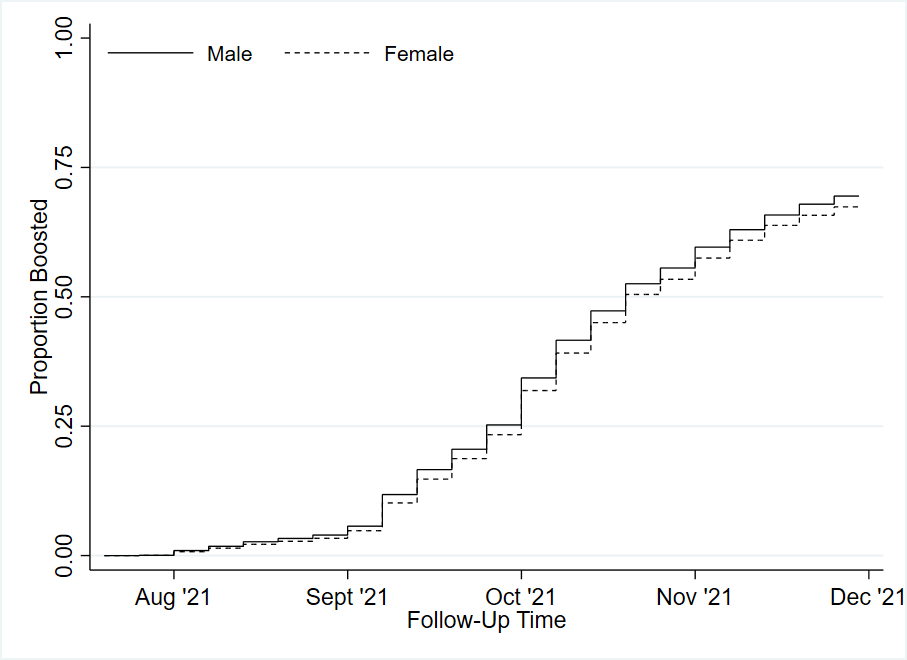


c)


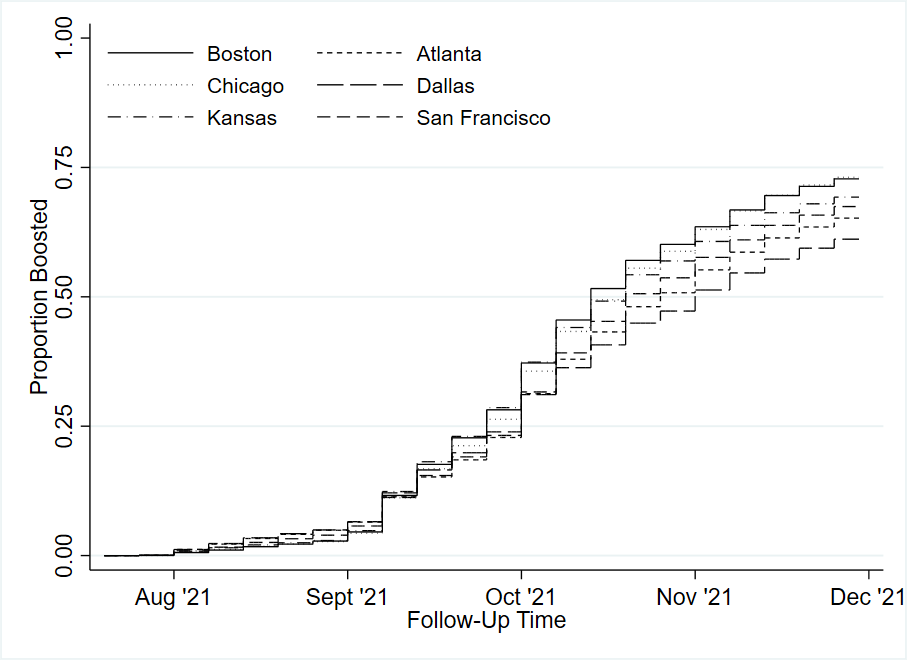


d)


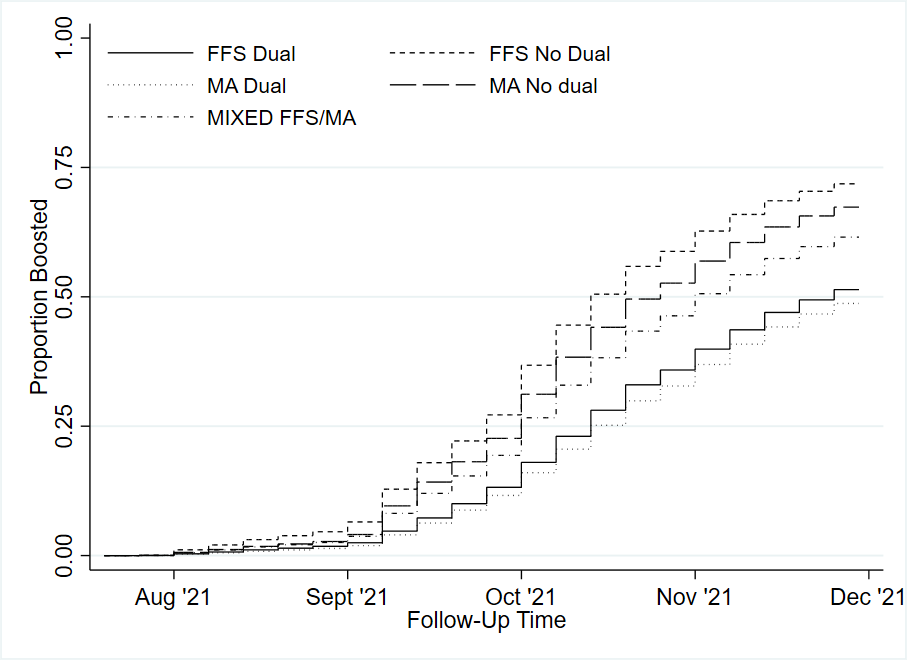


*Selected geographic regions only to enhance clarity of graph.

## Supplemental Figure 3. Kaplan-Meier cumulative incidence of booster vaccination when restricting to Medicare Fee-for-Service beneficiaries and extending follow-up to May 15, 2022 (n=6,059,015).


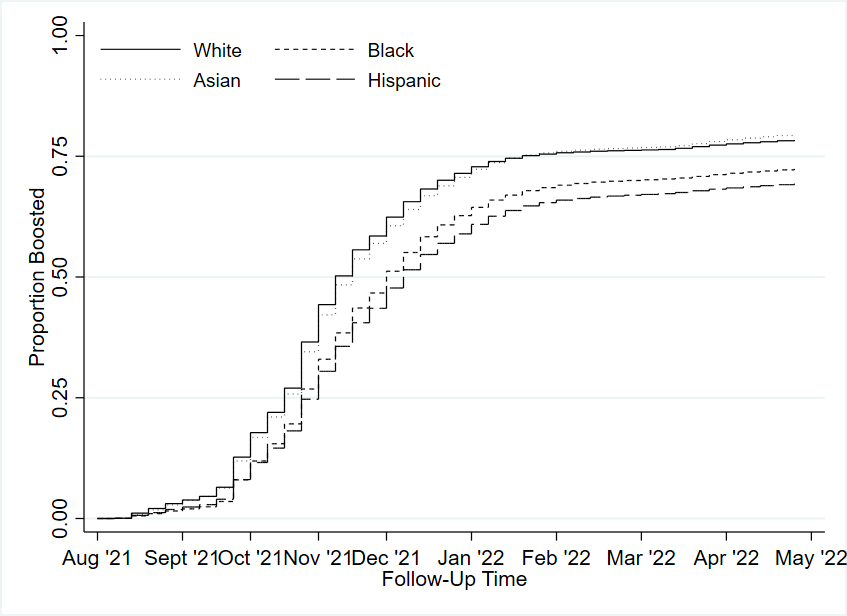


## Supplemental Figure 4. Relative rates with 95% confidence intervals of booster uptake by race and ethnicity, stratified by a) sex and b) age group.

b)

## Supplemental Figure 5. Relative rates with 95% confidence intervals of booster uptake by race and ethnicity stratified degree of urbanicity.

# Supplementary Tables

## Supplementary Table 1. Common Procedural Terminology (CPT) Codes used to Identify COVID-19 Vaccines.

| **Vaccine Type** | **Codes** |
| --- | --- |
| BNT162b2, Dose 1 | 0001A |
| mRNA-1273, Dose 1 | 0011A |
| JNJ-78436735 | 0034A |
| BNT162b2, Dose 2 | 0002A |
| mRNA-1273, Dose 2 | 0012A |
| BNT162b2, Dose 3 | 0003A |
| BNT162b2, Booster Dose | 0004A |
| BNT162b2, Booster Dose | 0124A |
| mRNA-1273, Dose 3 | 0013A |
| mRNA-1273, Booster Dose | 0064A |

*NB: Both Dose 3 and Booster Dose were considered COVID-19 booster vaccine doses in the present study.*

## Supplemental Table 2. Characteristics of all community-dwelling Medicare beneficiaries 66 years and older with 2 documented mRNA vaccine doses as of August 1, 2021.

| **Characteristics** | **Overall** | **White Race** | **Black Race** | **Asian Race** | **Hispanic Ethnicity** | **Native American Race** | **Other Race** | **Unknown or Missing Race/Ethnicity** |
| --- | --- | --- | --- | --- | --- | --- | --- | --- |
| **Unique Beneficiaries, n (%)** | 11,339,103 (100.0%) | 8,863,202 (100.0%) | 855,319 (100.0%) | 461,650 (100.0%) | 773,790 (100.0%) | 17,034 (100.0%) | 111,190 (100.0%) | 256,918 (100.0%) |
| **Age in years, mean (SD)** | 76.20 (7.36) | 76.51 (7.47) | 75.04 (6.85) | 75.97 (7.31) | 75.71 (6.94) | 76.05 (7.01) | 76.13 (6.79) | 71.44 (3.35) |
| **Age in years, n (%)** |  |  |  |  |  |  |  |  |
| 66-69 | 2,643,312 (23.31%) | 1,987,430 (22.42%) | 237,473 (27.76%) | 111,807 (24.22%) | 188,880 (24.41%) | 3,691 (21.67%) | 24,128 (21.70%) | 89,903 (34.99%) |
| 70-74 | 3,288,099 (29.00%) | 2,481,305 (28.00%) | 263,453 (30.80%) | 136,167 (29.50%) | 229,065 (29.60%) | 5,352 (31.42%) | 30,514 (27.44%) | 142,243 (55.37%) |
| 75-79 | 2,292,169 (20.21%) | 1,827,399 (20.62%) | 163,665 (19.13%) | 91,065 (19.73%) | 160,065 (20.69%) | 3,476 (20.41%) | 26,079 (23.45%) | 20,420 (7.95%) |
| 80-84 | 1,532,112 (13.51%) | 1,236,918 (13.96%) | 104,921 (12.27%) | 61,677 (13.36%) | 105,951 (13.69%) | 2,391 (14.04%) | 17,757 (15.97%) | 2,497 (0.97%) |
| 85-89 | 933,860 (8.24%) | 772,809 (8.72%) | 55,443 (6.48%) | 35,843 (7.76%) | 58,061 (7.50%) | 1,317 (7.73%) | 9,222 (8.29%) | 1,165 (0.45%) |
| 90+ | 649,551 (5.73%) | 557,341 (6.29%) | 30,364 (3.55%) | 25,091 (5.44%) | 31,768 (4.11%) | 807 (4.74%) | 3,490 (3.14%) | 690 (0.27%) |
| **Female sex , n(%)** | 6,746,330 (59.50%) | 5,280,685 (59.58%) | 554,109 (64.78%) | 270,119 (58.51%) | 468,629 (60.56%) | 11,137 (65.38%) | 60,870 (54.74%) | 100,781 (39.23%) |
| **Geographic Region, n(%)** |  |  |  |  |  |  |  |  |
| Boston | 785,317 (6.93%) | 678,063 (7.65%) | 24,735 (2.89%) | 20,879 (4.52%) | 27,635 (3.57%) | 252 (1.48%) | 6,441 (5.79%) | 27,312 (10.63%) |
| New York | 1,281,419 (11.30%) | 873,281 (9.85%) | 81,177 (9.49%) | 66,442 (14.39%) | 209,633 (27.09%) | 730 (4.29%) | 14,877 (13.38%) | 35,279 (13.73%) |
| Philadelphia | 1,045,382 (9.22%) | 856,749 (9.67%) | 110,113 (12.87%) | 27,883 (6.04%) | 17,970 (2.32%) | 255 (1.50%) | 7,336 (6.60%) | 25,076 (9.76%) |
| Atlanta | 2,226,798 (19.64%) | 1,737,900 (19.61%) | 290,320 (33.94%) | 32,485 (7.04%) | 110,504 (14.28%) | 1,756 (10.31%) | 12,530 (11.27%) | 41,303 (16.08%) |
| Chicago | 2,419,196 (21.33%) | 2,095,549 (23.64%) | 143,936 (16.83%) | 46,195 (10.01%) | 53,306 (6.89%) | 1,773 (10.41%) | 15,095 (13.58%) | 63,342 (24.65%) |
| Dallas | 927,542 (8.18%) | 648,520 (7.32%) | 106,915 (12.50%) | 27,819 (6.03%) | 118,475 (15.31%) | 6,265 (36.78%) | 6,407 (5.76%) | 13,141 (5.11%) |
| Kansas City | 429,773 (3.79%) | 387,007 (4.37%) | 22,583 (2.64%) | 4,647 (1.01%) | 5,561 (0.72%) | 481 (2.82%) | 1,755 (1.58%) | 7,739 (3.01%) |
| Denver | 200,250 (1.77%) | 179,310 (2.02%) | 2,315 (0.27%) | 2,936 (0.64%) | 8,746 (1.13%) | 725 (4.26%) | 1,234 (1.11%) | 4,984 (1.94%) |
| San Francisco | 1,639,651 (14.46%) | 1,067,698 (12.05%) | 61,024 (7.13%) | 220,747 (47.82%) | 213,640 (27.61%) | 3,161 (18.56%) | 42,438 (38.17%) | 30,943 (12.04%) |
| Seattle | 303,383 (2.68%) | 270,147 (3.05%) | 3,730 (0.44%) | 10,966 (2.38%) | 7,358 (0.95%) | 1,428 (8.38%) | 2,852 (2.56%) | 6,902 (2.69%) |
| Missing | 80,392 (0.71%) | 68,978 (0.78%) | 8,471 (0.99%) | 651 (0.14%) | 962 (0.12%) | 208 (1.22%) | 225 (0.20%) | 897 (0.35%) |
| **Social deprivation index, n(%)** |  |  |  |  |  |  |  |  |
| Quintile 1 (low deprivation) | 2,213,826 (19.52%) | 1,959,358 (22.11%) | 46,729 (5.46%) | 74,134 (16.06%) | 43,199 (5.58%) | 1,264 (7.42%) | 20,781 (18.69%) | 68,361 (26.61%) |
| Quintile 2 | 2,230,557 (19.67%) | 1,937,061 (21.86%) | 70,958 (8.30%) | 77,678 (16.83%) | 64,135 (8.29%) | 2,171 (12.75%) | 21,234 (19.10%) | 57,320 (22.31%) |
| Quintile 3 | 2,178,134 (19.21%) | 1,819,937 (20.53%) | 101,818 (11.90%) | 94,930 (20.56%) | 86,036 (11.12%) | 2,881 (16.91%) | 23,019 (20.70%) | 49,513 (19.27%) |
| Quintile 4 | 2,208,971 (19.48%) | 1,758,184 (19.84%) | 169,682 (19.84%) | 86,494 (18.74%) | 125,931 (16.27%) | 3,968 (23.29%) | 21,116 (18.99%) | 43,596 (16.97%) |
| Quintile 5 (high deprivation) | 2,240,698 (19.76%) | 1,284,306 (14.49%) | 448,075 (52.39%) | 124,535 (26.98%) | 318,690 (41.19%) | 6,324 (37.13%) | 23,567 (21.20%) | 35,201 (13.70%) |
| Missing | 266,917 (2.35%) | 104,356 (1.18%) | 18,057 (2.11%) | 3,879 (0.84%) | 135,799 (17.55%) | 426 (2.50%) | 1,473 (1.32%) | 2,927 (1.14%) |
| **Urbanicity, n(%)** |  |  |  |  |  |  |  |  |
| Large central metro | 3,084,935 (27.21%) | 2,056,587 (23.20%) | 352,136 (41.17%) | 228,372 (49.47%) | 330,496 (42.71%) | 3,143 (18.45%) | 42,919 (38.60%) | 71,282 (27.75%) |
| Large fringe metro | 3,240,539 (28.58%) | 2,655,316 (29.96%) | 209,295 (24.47%) | 123,226 (26.69%) | 136,585 (17.65%) | 2,088 (12.26%) | 31,169 (28.03%) | 82,860 (32.25%) |
| Medium metro | 2,605,362 (22.98%) | 2,134,496 (24.08%) | 166,955 (19.52%) | 87,958 (19.05%) | 127,535 (16.48%) | 4,479 (26.29%) | 25,256 (22.71%) | 58,683 (22.84%) |
| Small metro | 1,018,903 (8.99%) | 895,694 (10.11%) | 54,580 (6.38%) | 11,348 (2.46%) | 29,395 (3.80%) | 2,186 (12.83%) | 5,159 (4.64%) | 20,541 (8.00%) |
| Micropolitan | 802,856 (7.08%) | 709,225 (8.00%) | 41,954 (4.91%) | 9,291 (2.01%) | 18,515 (2.39%) | 3,471 (20.38%) | 5,027 (4.52%) | 15,373 (5.98%) |
| Non-core | 457,209 (4.03%) | 409,048 (4.62%) | 30,195 (3.53%) | 1,351 (0.29%) | 5,350 (0.69%) | Suppressed* | 1,472 (1.32%) | 8,128 (3.16%) |
| Missing | 129,299 (1.14%) | 2,836 (0.03%) | 204 (0.02%) | 104 (0.02%) | 125,914 (16.27%) | Suppressed* | 188 (0.17%) | 51 (0.02%) |
| **Dual Enrollment** |  |  |  |  |  |  |  |  |
| Full Dual | 824,795 (7.27%) | 357,445 (4.03%) | 131,129 (15.33%) | 132,505 (28.70%) | 172,188 (22.25%) | 3,513 (20.62%) | 10,048 (9.04%) | 17,967 (6.99%) |
| FFS with no Medicaid dual enrollment | 5,706,585 (50.33%) | 4,917,746 (55.48%) | 270,478 (31.62%) | 147,836 (32.02%) | 162,221 (20.96%) | 7,696 (45.18%) | 52,532 (47.25%) | 148,076 (57.64%) |
| FFS with Medicaid dual enrollment | 352,430 (3.11%) | 176,434 (1.99%) | 41,808 (4.89%) | 61,932 (13.42%) | 56,116 (7.25%) | 1,951 (11.45%) | 4,857 (4.37%) | 9,332 (3.63%) |
| MA with no Medicaid dual enrollment | 4,426,011 (39.03%) | 3,301,840 (37.25%) | 408,265 (47.73%) | 167,787 (36.35%) | 415,436 (53.69%) | 4,923 (28.90%) | 44,500 (40.02%) | 83,260 (32.41%) |
| MA with Medicaid dual enrollment | 403,358 (3.56%) | 151,294 (1.71%) | 75,391 (8.81%) | 61,617 (13.35%) | 102,746 (13.28%) | 1,150 (6.75%) | 4,408 (3.96%) | 6,752 (2.63%) |
| MIXED with no Medicaid dual enrollment | 381,712 (3.37%) | 286,171 (3.23%) | 45,447 (5.31%) | 13,522 (2.93%) | 23,945 (3.09%) | 902 (5.30%) | 4,110 (3.70%) | 7,615 (2.96%) |
| MIXED with Medicaid dual enrollment | 69,007 (0.61%) | 29,717 (0.34%) | 13,930 (1.63%) | 8,956 (1.94%) | 13,326 (1.72%) | 412 (2.42%) | 783 (0.70%) | 1,883 (0.73%) |
| **Medicare Enrollment** |  |  |  |  |  |  |  |  |
| FFS Beneficiaries Only | 6,059,015 (53.43%) | 5,094,180 (57.48%) | 312,286 (36.51%) | 209,768 (45.44%) | 218,337 (28.22%) | 9,647 (56.63%) | 57,389 (51.61%) | 157,408 (61.27%) |
| MA Beneficiaries Only | 4,829,369 (42.59%) | 3,453,134 (38.96%) | 483,656 (56.55%) | 229,404 (49.69%) | 518,182 (66.97%) | 6,073 (35.65%) | 48,908 (43.99%) | 90,012 (35.04%) |
| MIXED Beneficiaries | 450,719 (3.97%) | 315,888 (3.56%) | 59,377 (6.94%) | 22,478 (4.87%) | 37,271 (4.82%) | 1,314 (7.71%) | 4,893 (4.40%) | 9,498 (3.70%) |
| **History of Comorbidities*** |  |  |  |  |  |  |  |  |
| Cancer | 953,327 (15.73%) | 814,447 (15.99%) | 51,868 (16.61%) | 24,659 (11.76%) | 28,413 (13.01%) | 1,252 (12.98%) | 8,181 (14.26%) | 24,507 (15.57%) |
| Chronic Obstructive Pulmonary Disease | 640,770 (10.58%) | 560,042 (10.99%) | 34,117 (10.92%) | 12,991 (6.19%) | 19,232 (8.81%) | 1,529 (15.85%) | 3,987 (6.95%) | 8,872 (5.64%) |
| Congestive Heart Failure | 765,781 (12.64%) | 648,310 (12.73%) | 51,271 (16.42%) | 19,384 (9.24%) | 28,110 (12.87%) | 1,622 (16.81%) | 6,033 (10.51%) | 11,051 (7.02%) |
| Diabetes | 1,498,904 (24.74%) | 1,154,815 (22.67%) | 129,745 (41.55%) | 75,568 (36.02%) | 83,877 (38.42%) | 4,039 (41.87%) | 19,476 (33.94%) | 31,384 (19.94%) |
| Ischemic Stroke | 177,035 (2.92%) | 146,733 (2.88%) | 13,137 (4.21%) | 5,520 (2.63%) | 6,949 (3.18%) | 380 (3.94%) | 1,606 (2.80%) | 2,710 (1.72%) |
| Major Organ Transplant | 22,189 (0.37%) | 17,884 (0.35%) | 1,426 (0.46%) | 753 (0.36%) | 1,130 (0.52%) | 53 (0.55%) | 222 (0.39%) | 721 (0.46%) |
| Vasular disease | 1,174,801 (19.39%) | 1,000,709 (19.64%) | 68,260 (21.86%) | 31,134 (14.84%) | 42,846 (19.62%) | 1,905 (19.75%) | 9,624 (16.77%) | 20,323 (12.91%) |
| Renal Conditions | 350,354 (5.78%) | 288,327 (5.66%) | 28,990 (9.28%) | 10,145 (4.84%) | 14,101 (6.46%) | 840 (8.71%) | 3,054 (5.32%) | 4,897 (3.11%) |
| **Cumulative Frailty Index*** |  |  |  |  |  |  |  |  |
| Non-frail | 3,336,657 (55.07%) | 2,771,470 (54.40%) | 161,265 (51.64%) | 134,691 (64.21%) | 116,279 (53.26%) | 3,994 (41.40%) | 35,063 (61.10%) | 113,895 (72.36%) |
| Pre-Frail | 2,329,921 (38.45%) | 1,980,852 (38.88%) | 129,803 (41.57%) | 67,480 (32.17%) | 87,189 (39.93%) | 4,669 (48.40%) | 19,685 (34.30%) | 40,243 (25.57%) |
| Frail | 392,437 (6.48%) | 341,858 (6.71%) | 21,218 (6.79%) | 7,597 (3.62%) | 14,869 (6.81%) | 984 (10.20%) | 2,641 (4.60%) | 3,270 (2.08%) |
| **Combined Comorbidity Index, mean (SD)*** | 1.73 (2.65) | 1.73 (2.65) | 2.10 (2.98) | 1.41 (2.47) | 1.81 (2.75) | 2.32 (2.96) | 1.54 (2.55) | 1.07 (2.16) |

**To avoid back-calculation of small cells (<25).*

## Supplemental Table 3. Number of beneficiaries with booster vaccine receipt, death, and censoring events by race and ethnicity group.

| **Group** | **N (Total Unique Beneficiaries)** | **Events** | | **Died** | | **Censored due to disenrollment** | | **Average follow-up time in weeks, mean (SD)** |
| --- | --- | --- | --- | --- | --- | --- | --- | --- |
|  |  | **#** | **%** | **#** | **%** | **#** | **%** |  |
| All | 11,339,103 | 7,649,945 | 67.47% | 472,903 | 4.17% | 1809 | 0.02% | 15.5 (5.4) |
| White | 8,863,202 | 6,129,769 | 69.16% | 395,322 | 4.46% | 1153 | 0.01% | 15.2 (5.4) |
| Black | 855,319 | 499,875 | 58.44% | 32,117 | 3.75% | 186 | 0.02% | 16.8 (5.1) |
| Other | 111,190 | 77,988 | 70.14% | 3,432 | 3.09% | 32 | 0.03% | 15.2 (5.4) |
| Asian | 461,650 | 315,744 | 68.39% | 11,952 | 2.59% | 163 | 0.04% | 15.6 (5.4) |
| Hispanic | 773,790 | 425,396 | 54.98% | 25,411 | 3.28% | 179 | 0.02% | 17.2 (5.1) |
| Unknown/Missing | 256,918 | 191,851 | 74.67% | 3,677 | 1.43% | suppressed * | suppressed * | 14.6 (5.4) |
| Native American | 17,034 | 9,322 | 54.73% | 992 | 5.82% | suppressed * | suppressed * | 16.6 (5.6) |

**To avoid back-calculation of small cells (<25).*

## Supplemental Table 4. Results from crude, partially-adjusted, and fully-adjusted models comparing receipt of booster vaccinations by race and ethnicity.

| **Model 1** | | | **Model 2** | | | **Model 3** | | |
| --- | --- | --- | --- | --- | --- | --- | --- | --- |
| **Contrast** | **Estimate** | **95% CI** | **Contrast** | **Estimate** | **95% CI** | **Contrast** | **Estimate** | **95% CI** |
| Black (vs. White) | 0.7646 | (0.7624, 0.7668) | Black (vs. White) | 0.7626 | (0.7604, 0.7648) | Black (vs. White) | 0.7779 | (0.7756, 0.7802) |
| Asian | 0.9674 | (0.9640, 0.9709) | Asian | 0.9656 | (0.9621, 0.9690) | Asian | 0.9715 | (0.9680, 0.9751) |
| Hispanic | 0.7048 | (0.7026, 0.7070) | Hispanic | 0.7020 | (0.6998, 0.7042) | Hispanic | 0.7210 | (0.7187, 0.7233) |
| Native American | 0.7262 | (0.7116, 0.7412) | Native American | 0.7245 | (0.7100, 0.7394) | Native American | 0.7574 | (0.7421, 0.7729) |
| Other | 1.0174 | (1.0103, 1.0246) | Other | 1.0099 | (1.0028, 1.0171) | Other | 1.0142 | (1.0070, 1.0214) |
| Missing/unknown | 1.1282 | (1.1231, 1.1334) | Missing/unknown | 1.1026 | (1.0975, 1.1076) | Missing/unknown | 1.0944 | (1.0894, 1.0995) |
|  |  |  | Male (vs. Female) | 1.0379 | (1.0364, 1.0394) | Male (vs. Female) | 1.0379 | (1.0364, 1.0394) |
|  |  |  | 70 to <75 (vs. 66 to <70) | 1.0648 | (1.0627, 1.0669) | 70 to <75 (vs. 66 to <70) | 1.0647 | (1.0626, 1.0668) |
|  |  |  | 75 to <80 | 1.0682 | (1.0659, 1.0705) | 75 to <80 | 1.0679 | (1.0656, 1.0702) |
|  |  |  | 80 to <85 | 1.0281 | (1.0256, 1.0306) | 80 to <85 | 1.0271 | (1.0246, 1.0296) |
|  |  |  | 85 to <90 | 0.9615 | (0.9587, 0.9643) | 85 to <90 | 0.9599 | (0.9571, 0.9627) |
|  |  |  | 90+ | 0.8792 | (0.8761, 0.8823) | 90+ | 0.8766 | (0.8736, 0.8797) |
|  |  |  |  |  |  | New York (vs Boston) | 0.8993 | (0.8963, 0.9024) |
|  |  |  |  |  |  | Philadelphia | 0.9346 | (0.9313, 0.9379) |
|  |  |  |  |  |  | Atlanta | 0.8727 | (0.87, 0.8754) |
|  |  |  |  |  |  | Chicago | 0.9972 | (0.9942, 1.0002) |
|  |  |  |  |  |  | Dallas | 0.8242 | (0.8211, 0.8272) |
|  |  |  |  |  |  | Kansas City | 0.9384 | (0.9342, 0.9426) |
|  |  |  |  |  |  | Denver | 0.8954 | (0.8901, 0.9008) |
|  |  |  |  |  |  | San Francisco | 0.9213 | (0.9183, 0.9243) |
|  |  |  |  |  |  | Seattle | 0.8918 | (0.8872, 0.8963) |
|  |  |  |  |  |  | Missing or Other | 0.8639 | (0.8562, 0.8717) |

## Supplemental Table 5. Results from sensitivity analysis including Medicare and Medicaid enrollment type in the regression models comparing booster vaccine receipt.

| **Contrast** | **Relative Rate** | **95% CI** |
| --- | --- | --- |
| Black (vs. White) | 0.8308 | (0.8284, 0.8333) |
| Asian | 1.0784 | (1.0744, 1.0824) |
| Hispanic | 0.7916 | (0.7891, 0.7942) |
| Native American | 0.8094 | (0.7931, 0.826) |
| Other | 1.0379 | (1.0306, 1.0453) |
| Missing/unknown | 1.1067 | (1.1016, 1.1118) |
| Male (vs. Female) | 1.0309 | (1.0294, 1.0324) |
| 70 to <75 (vs. 66 to <70) | 1.0579 | (1.0558, 1.0599) |
| 75 to <80 | 1.0631 | (1.0608, 1.0654) |
| 80 to <85 | 1.0249 | (1.0224, 1.0274) |
| 85 to <90 | 0.9597 | (0.9569, 0.9625) |
| 90+ | 0.8750 | (0.8719, 0.8780) |
| New York (vs Boston) | 0.8925 | (0.8895, 0.8955) |
| Philadelphia | 0.9129 | (0.9097, 0.9161) |
| Atlanta | 0.8615 | (0.8588, 0.8641) |
| Chicago | 0.9937 | (0.9908, 0.9967) |
| Dallas | 0.8100 | (0.8070, 0.8130) |
| Kansas City | 0.9263 | (0.9222, 0.9305) |
| Denver | 0.8873 | (0.882, 0.8926) |
| San Francisco | 0.9243 | (0.9213, 0.9273) |
| Seattle | 0.8978 | (0.8932, 0.9024) |
| Missing or Other | 0.8471 | (0.8395, 0.8547) |
| FFS with dual Medicaid enrollment (vs. FFS without dual Medicaid enrollment) | 0.6223 | (0.6193, 0.6253) |
| Medicare Advantage with dual Medicaid enrollment | 0.6031 | (0.6002, 0.6059) |
| Medicare Advantage without dual Medicaid enrollment | 0.9109 | (0.9095, 0.9123) |
| Mixed FFS/MA Enrollment with or without Medicaid enrollment | 0.8047 | (0.8016, 0.8079) |
| **Notes:** CI=confidence interval; FFS=fee-for-service; MA=Medicare Advantage | | |

## Supplemental Table 6. Results from sensitivity analysis restricting to Fee-for-Service Medicare beneficiaries and extending follow-up to May 15, 2022 (n=6,059,015).

| **Contrast** | **Relative Rate** | **95% CI** |
| --- | --- | --- |
| Black (vs. White) | 0.8207 | (0.8172,0.8243) |
| Asian | 0.9986 | (0.9936, 1.0036) |
| Hispanic | 0.7549 | (0.7510,0.7589) |
| Native American | 0.7579 | (0.7394, 0.7770) |
| Other | 1.0388 | (1.0293, 1.0485) |
| Missing/unknown | 1.1051 | (1.0293,1.0485) |
| Male (vs. Female) | 1.0336 | (1.0316,1.0355) |
| 70 to <75 (vs. 66 to <70) | 1.0642 | (1.0616, 1.0669) |
| 75 to <80 | 1.0662 | (1.0632, 1.0691) |
| 80 to <85 | 1.0169 | (1.0137, 1.0201) |
| 85 to <90 | 0.9354 | (0.9319, 0.9389) |
| 90+ | 0.8358 | (0.8321, 0.8394) |
| New York (vs Boston) | 0.9111 | (0.9072, 0.9150) |
| Philadelphia | 0.9079 | (0.9041, 0.9117) |
| Atlanta | 0.8528 | (0.8495, 0.8561) |
| Chicago | 0.9707 | (0.9671, 0.9744) |
| Dallas | 0.7747 | (0.7711, 0.7784) |
| Kansas City | 0.9230 | (0.9179, 0.9281) |
| Denver | 0.8294 | (0.8230, 0.8358) |
| San Francisco | 0.9238 | (0.9200, 0.9275) |
| Seattle | 0.8078 | (0.8022, 0.8134) |
| Missing or Other | 0.8339 | (0.8252, 0.8428) |

## Supplemental Table 7. Absolute booster vaccine uptake by race and ethnicity group across region, urbanicity, and Medicare enrollment

|  | **Cumulative proportion with a booster vaccine (Kaplan-Meier estimates)** | | | |
| --- | --- | --- | --- | --- |
|  | **White** | **Black** | **Asian** | **Hispanic** |
| **Geographic region** |  |  |  |  |
| Boston | 72.73% | 55.14% | 67.56% | 51.00% |
| New York | 69.03% | 52.00% | 63.95% | 51.76% |
| Philadelphia | 69.47% | 57.76% | 69.10% | 57.03% |
| Atlanta | 64.91% | 57.50% | 65.62% | 52.83% |
| Chicago | 72.66% | 57.51% | 71.57% | 61.04% |
| Dallas | 60.75% | 57.59% | 64.74% | 51.42% |
| Kansas City | 68.68% | 56.29% | 66.19% | 60.22% |
| Denver | 65.80% | 50.68% | 63.17% | 52.23% |
| San Francisco | 68.00% | 58.24% | 67.33% | 53.83% |
| Seattle | 66.30% | 56.46% | 64.57% | 57.43% |
| Other | 65.38% | 58.73% | 66.55% | 47.92% |
| **Urbanicity** |  |  |  |  |
| Large central metro | 70.10% | 55.05% | 64.84% | 52.47% |
| Large fringe metro | 69.82% | 59.43% | 69.63% | 55.62% |
| Medium metro | 68.99% | 58.46% | 70.40% | 53.52% |
| Small metro | 66.41% | 56.14% | 65.21% | 53.59% |
| Micropolitan | 64.08% | 56.72% | 56.54% | 52.56% |
| Non-core | 61.39% | 55.85% | 62.33% | 51.93% |
| **Medicare enrollment** |  |  |  |  |
| FFS with no Medicaid dual enrollment | 70.75% | 63.53% | 73.62% | 61.39% |
| FFS with Medicaid dual enrollment | 49.91% | 43.00% | 57.50% | 44.15% |
| MA with no Medicaid dual enrollment | 67.58% | 57.74% | 69.38% | 54.50% |
| MA with Medicaid dual enrollment | 48.17% | 41.27% | 56.39% | 42.59% |
| Mixed FFS/MA | 62.44% | 51.28% | 61.02% | 47.63% |
| **Notes:** FFS=fee-for-service; MA=Medicare Advantage; Estimates reflect uptake at the intersection of the row (subgroups) and column. For example, 72.73% of White individuals living in the Boston region received a booster vaccine compared to 55.14% of Black individuals living in the Boston region. | | | | |

# References

1. HHS Regional Offices. [cited 2023 Apr 10]; Available from: https://www.hhs.gov/about/agencies/iea/regional-offices/index.html

2. Social Deprivation Index (SDI). [cited 2022 Nov 29]; Available from: https://www.graham-center.org/content/brand/rgc/maps-data-tools/social-deprivation-index.html

3. Dually Eligible Individuals - Categories. [cited 2023 Mar 5]; Available from: https://www.cms.gov/Medicare-Medicaid-Coordination/Medicare-and-Medicaid-Coordination/Medicare-Medicaid-Coordination-Office/Downloads/MedicareMedicaidEnrolleeCategories.pdf

4. Risk Adjustment | CMS. [cited 2022 Dec 8]; Available from: https://www.cms.gov/Medicare/Health-Plans/MedicareAdvtgSpecRateStats/Risk-Adjustors

5. Kim DH, Schneeweiss S. Measuring frailty using claims data for pharmacoepidemiologic studies of mortality in older adults: evidence and recommendations: FRAILTY INDEX IN CLAIMS DATABASE. Pharmacoepidemiol Drug Saf. 2014 Sep;23(9):891–901.

6. Gagne JJ, Glynn RJ, Avorn J, Levin R, Schneeweiss S. A combined comorbidity score predicted mortality in elderly patients better than existing scores. J Clin Epidemiol. 2011 Jul;64(7):749–59.

7. Sun JW, Rogers JR, Her Q, Welch EC, Panozzo CA, Toh S, et al. Adaptation and Validation of the Combined Comorbidity Score for ICD-10-CM. Med Care. 2017 Dec;55(12):1046–51.
